# Advances in the Treatment of Autosomal Dominant Polycystic Kidney Disease and Novel Therapeutic Targets

**DOI:** 10.3390/cimb48050468

**Published:** 2026-04-30

**Authors:** Wenzheng Zhang, Tianze Sun, Xin Wang, Tao Jiang

**Affiliations:** School of Basic Medical Sciences, Beihua University, Jilin 132013, China; 18629963848@163.com (W.Z.); 18543276587@163.com (T.S.); 17671364399@163.com (X.W.)

**Keywords:** ADPKD, cAMP, tolvaptan, mTOR inhibitors, treatment, novel targets

## Abstract

Autosomal dominant polycystic kidney disease (ADPKD), with a prevalence of approximately 1 in 1000, is the most common inherited cause of end-stage renal disease (ESRD). It is primarily caused by mutations in the *PKD1* or *PKD2* genes. Multiple studies have demonstrated that deficiency of polycystin proteins, dysregulation of signaling pathways, and activation of inflammatory factors contribute to the progression of ADPKD. The cAMP-targeting drug tolvaptan is currently the only approved therapy for ADPKD; however, its side effects and high cost have limited its widespread use. Meanwhile, mTOR inhibitors, AMPK-targeting agents, anti-inflammatory agents, and dietary interventions have shown promising results in treating ADPKD. Furthermore, the emergence of novel targets such as Notch3 and AURKA offers new directions for ADPKD therapy. This article aims to review the pathogenesis of ADPKD and current treatment advances, while exploring potential new targets for future research, hoping to provide a scientific theoretical foundation for disease management.

## 1. Introduction

Autosomal dominant polycystic kidney disease (ADPKD) is the most common inherited renal disorder, with an estimated incidence ranging from 1 in 1000 to 1 in 2500 live births [1]. It is primarily caused by mutations in the PKD1 or PKD2 genes, which encode polycystin-1 (PC1) and polycystin-2 (PC2), respectively. These proteins are involved in regulating key cellular processes, including differentiation, proliferation, apoptosis, and cell adhesion [2]. The hallmark of ADPKD is the progressive development of numerous fluid-filled cysts within the kidneys, which ultimately disrupts normal renal architecture and function, leading to end-stage renal disease (ESRD). In addition to renal manifestations, ADPKD is associated with various extrarenal complications, such as hypertension, hepatic cysts, intracranial aneurysms, and cardiac valve abnormalities [3]. Although the condition follows an autosomal dominant inheritance pattern, cyst formation typically requires a somatic “second-hit” mutation that further reduces or abolishes the functional dosage of the corresponding polycystin in renal tubular or biliary epithelial cells [4]. Once formed, cyst expansion—driven by epithelial proliferation and fluid secretion—exerts pressure on the surrounding parenchyma, resulting in local injury, inflammation, and fibrosis. This review aims to synthesize recent advances in the pathophysiology, genetics, and clinical management of ADPKD, incorporating evidence from key trials, systematic reviews, and translational studies published in the past three years. We also highlight emerging therapeutic strategies beyond tolvaptan, including metabolic interventions, gene editing, and organoid-based disease modeling, to inform future research directions and clinical decision-making.

## 2. Pathogenesis of ADPKD

Mutations in *PKD1* (located on chromosome 16p13.3) account for approximately 78–85% of ADPKD cases, while mutations in *PKD2* (chromosome 4q22.1) are responsible for about 15–20% of cases. The remaining 5–10% of cases are genetically unresolved or attributed to rare mutations in other loci [5]. PC1 is a large integral membrane protein involved in the modulation of multiple signaling cascades. Its C-terminal tail mediates interaction with PC2, a transient receptor potential channel that regulates ion transport and indirectly influences intracellular Ca^2+^ signaling [6]. Both polycystins are primarily localized to the primary cilium—a microtubule-based sensory organelle that extends from the apical surface of renal epithelial cells into the tubular lumen. At the ciliary membrane, polycystins form multiprotein complexes that coordinate key signaling pathways—including Ca^2+^, cAMP, mTOR, Wnt, vascular endothelial growth factor (VEGF), and Hippo signaling—to establish and maintain the differentiated state of renal epithelial cells. Accumulating evidence supports a central role for the primary cilium in ADPKD pathogenesis [7,8,9]; notably, ciliary ablation in the setting of polycystin deficiency has been shown to suppress cyst growth [10]. As the disease progresses, reduced intracellular Ca^2+^ influx, elevated cAMP levels, and aberrant activation of the RAS–RAF–ERK pathway in renal epithelial cells are critical drivers of cyst expansion. Other signaling mechanisms implicated in disease progression include dysregulation of the MAPK/ERK pathway, heterotrimeric G proteins, mTOR, phosphoinositide 3-kinase (PI3K)/AKT, AMP-activated protein kinase (AMPK), nuclear factor of activated T-cells (NFAT), JAK/STAT, and nuclear factor-κB (NF-κB) [11]. In addition, cyst growth is accompanied by profound metabolic reprogramming, characterized by increased glucose uptake, enhanced aerobic glycolysis, and impaired fatty acid oxidation [12,13].

**Figure 1:**
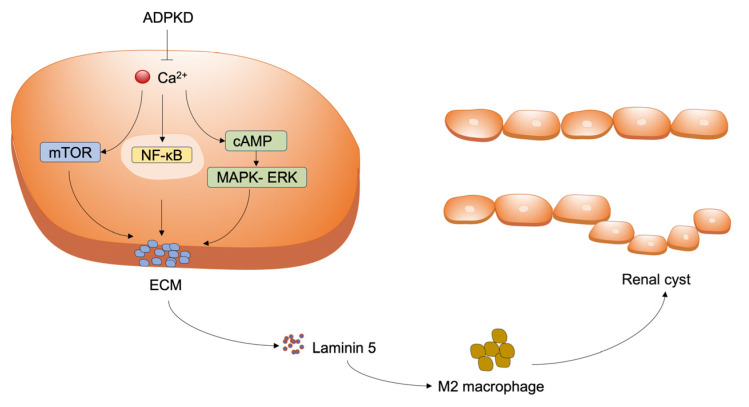
Pathogenesis of ADPKD. In ADPKD, polycystin deficiency leads to downregulation of intracellular calcium signaling, which releases the inhibition of the mTOR and NF-κB pathways, driving cystic epithelial cell proliferation and hypersecretion, and triggering basement membrane degradation and abnormal ECM deposition (including laminin 5 disorder). These changes recruit and polarize M2 macrophages, which further deteriorate the interstitial environment through pro-fibrotic effects, ultimately constituting a self-sustaining malignant cycle that drives the occurrence, expansion, and progression of renal cysts.

Accumulation of extracellular matrix (ECM) components and infiltration of inflammatory cells are observed in the early stages of ADPKD. In ADPKD kidneys, laminin-5 expression is upregulated within the ECM. Purified laminin-5 has been shown to activate extracellular signal-regulated kinase (ERK), thereby stimulating renal epithelial cell proliferation and promoting cystogenesis [14]. As cysts expand and compress the surrounding parenchyma and vasculature, obstructed glomeruli eventually give rise to atubular glomeruli, and apoptotic cell death becomes evident in proximal tubules [15]. Renal tubular epithelial cells, along with adjacent interstitial fibroblasts and infiltrating inflammatory cells, secrete a variety of chemokines, cytokines, and growth factors [16]. Among these, TGF-β promotes tubular cell dedifferentiation, recruits inflammatory cells to cystic sites, and activates α-smooth muscle actin (αSMA)-positive myofibroblasts, leading to increased ECM deposition. M2-like macrophages, in turn, secrete anti-inflammatory and pro-fibrotic mediators that stimulate tubular cell proliferation and myofibroblast activation. Pro-inflammatory stimuli and fibrotic accumulation also contribute to pericyte detachment, resulting in microvascular rarefaction and local tissue hypoxia, which further exacerbate the fibrotic response [1]. Pro-inflammatory molecules attract and activate more inflammatory cells, aggravating local injury and ultimately worsening cystic disease. The associated regulatory mechanism is given below (Figure 1).

## 3. Existing Pharmacological Therapies

### 3.1. Angiotensin-Converting Enzyme Inhibitors (ACEIs)

Current management of ADPKD primarily focuses on symptomatic control of hypertension and proteinuria, with particular attention to the evaluation and treatment of ADPKD-related complications, including cyst hemorrhage, cyst infection, nephrolithiasis, and chronic pain.

Cyst expansion in ADPKD compresses adjacent renal parenchyma and vasculature, leading to intrarenal ischemia and activation of the renin–angiotensin–aldosterone system (RAAS). This, in turn, increases aldosterone, promotes renal parenchymal fibrosis, and ultimately contributes to cyst enlargement and hypertension. Histological studies have found components of the RAAS—including angiotensinogen, renin, angiotensin-converting enzyme, angiotensin II, and angiotensin AT1 receptors—within cysts and dilated tubules of ADPKD kidneys, indicating a significant role for this system in the disease [17]. Strict blood pressure control, particularly via angiotensin blockade, remains a cornerstone of ADPKD management [1]. Recent studies show that ACEI treatment in ADPKD patients for 6 weeks significantly reduces renal vascular resistance and slows the rate of kidney disease progression [18]. However, ACEIs have potential side effects, including reduced renal blood flow leading to renal ischemia, hyperkalemia, and renal failure, necessitating judicious use and careful monitoring.

### 3.2. Vasopressin V2 Receptor Antagonists

Vasopressin acts on V2 receptors on the basolateral membrane of collecting duct cells to promote water reabsorption. Elevated vasopressin levels activate V2 receptors, increasing intracellular cyclic adenosine 3′,5′-monophosphate (cAMP), which influences cell proliferation and fluid secretion, thereby promoting cyst growth and renal function decline [19]. To date, vasopressin V2 receptor antagonists are the only therapy proven to have renoprotective effects [20].

#### 3.2.1. Tolvaptan

Historically, treatment for ADPKD was limited to symptom and complication management. Recently, the U.S. Food and Drug Administration (FDA) approved tolvaptan, the first pharmacotherapy indicated to slow the decline of kidney function in adults at risk of rapidly progressing ADPKD [21]. Tolvaptan is an oral, selective vasopressin V2 receptor antagonist [22] that reduces cAMP levels in the collecting duct and thick ascending limb [23] at cyst sites. By binding to V2 receptors on the vascular side of the collecting duct, it causes aquaporin-2 internalization from the apical membrane, blocking water reabsorption and increasing water excretion. This reduces the annual rate of kidney volume growth and slows renal function decline. Initial studies indicate that split daily dosing of tolvaptan is necessary to maintain effective V2 receptor inhibition, as evidenced by sustained suppression of urine osmolality to ~300 mOsm/kg [24], balancing efficacy and tolerability. Tolvaptan also shows benefit in patients with later-stage ADPKD [25]. Despite its approval, some patients experience adverse effects, such as polyuria and thirst, leading to intolerance [1]. In addition, a clinical trial with Somatostatin analogue Octreotide and Tolvaptan combinations has also been conducted. In patients with ADPKD, octreotide-LAR added-on tolvaptan reduced GFR effectively. Octreotide-LAR also reduced total and cystic kidney volumes and attenuated the aquaretic effect of tolvaptan [26].

#### 3.2.2. Lixivaptan

Lixivaptan is a potent, selective vasopressin V2 receptor antagonist. Wang et al. [27] fed ADPKD rats a lixivaptan-containing diet for 8 weeks and found a >50% reduction in cyst volume, a 23% decrease in intracellular cAMP levels compared to controls, and preliminary in vitro data suggesting a lower risk of hepatotoxicity than tolvaptan. These results provide the first evidence of lixivaptan’s potential therapeutic effect in ADPKD.

#### 3.2.3. Somatostatin Analogues

In ADPKD, mutations in PKD1 or PKD2 disrupt the normal differentiated phenotype of tubular epithelial cells, leading to increased intracellular cAMP. cAMP signaling plays a central role in ADPKD pathophysiology; its upregulation activates protein kinase A (PKA), driving cell proliferation via the cystic fibrosis transmembrane conductance regulator (CFTR) and promoting cystogenesis [28]. cAMP and PKA also lead to activation of the mitogen-activated protein kinase (MAPK) cascade and mammalian target of rapamycin (mTOR) [29,30], Wnt-dependent microtubule formation [31], increased cilium length [32], and centrosome amplification [33]. Cyst growth displaces and destroys normal kidney tissue, leading to renal function decline. Given the key role of elevated intracellular cAMP, therapies aimed at reducing cAMP have been investigated.

Somatostatin binds to five G protein-coupled receptors, directly or indirectly inhibiting cAMP production in hepatic and renal cells. This inhibition suppresses fluid secretion, cell proliferation, and can induce apoptosis. Natural somatostatin has a very short in vivo half-life and is rapidly cleared. Therefore, longer-acting synthetic analogues have been developed, such as octreotide, lanreotide, and pasireotide [34].

### 3.3. mTOR Inhibitors

mTOR is a serine/threonine protein kinase of the PI3K-related kinase (PIKK) family and the catalytic subunit of the protein complexes mTORC1 and mTORC2. It integrates signals from growth factors, ATP, oxygen, and amino acids to regulate cell growth and metabolism. PC-1 inhibits mTORC1 via interaction with the protein TSC2 [35]. In ADPKD, loss of this regulation results in hyperactive mTORC1 signaling, increasing protein translation and leading to cell proliferation, growth, and cystogenesis [35,36]. Rapamycin, a macrolide, exerts antiproliferative, growth-inhibitory, and antifibrotic effects by inhibiting the mTOR enzyme [37]. Experimental inhibition of mTOR with rapamycin slows PKD progression in rats. Sirolimus, a natural macrolide antibiotic produced by Streptomyces hygroscopicus, is a specific inhibitor of the mTOR pathway [38]. Sirolimus significantly delays disease progression in Han:SPRD (cy) rats, reducing proliferation in cystic and non-cystic tubules, inhibiting kidney enlargement and cyst formation, and preserving renal function. However, the effective dose in Pkd1 mutant rats far exceeds what is safely tolerable in humans [39], and adverse effects such as chronic proteinuria and hyperlipidemia are concerns.

### 3.4. AMPK Agonists

mTOR and AMPK are opposing energy metabolism sensors regulating cell growth and proliferation. AMPK is a key cellular sensor and regulator of energy homeostasis, highly expressed in the kidney and modulating various physiological and pathological processes [40,41]. It plays a central pathogenic role in conditions like obesity, metabolic syndrome, diabetes, inflammation, and cancer [42]. ADPKD is characterized by increased aerobic glycolysis, where glucose is preferentially converted to lactate rather than being fully oxidized in mitochondria [43]. In ADPKD mouse models, moderate dietary restriction slows cyst growth by activating AMPK and inhibiting mTOR signaling [43]. Therapeutic AMPK activation can mitigate the severity of cystic kidney disease by improving mitochondrial biogenesis and reducing tissue inflammation [22].

#### 3.4.1. Metformin

Type 2 diabetes medications like pioglitazone and metformin have recently shown positive effects in ADPKD. However, pioglitazone appears effective only when combined with tolvaptan [44]. Metformin, a biguanide, activates AMPK, which phosphorylates and inhibits CFTR, thereby inhibiting fluid and electrolyte secretion in epithelial cells [45]. Furthermore, AMPK phosphorylates tuberin, indirectly inhibiting the mTOR pathway. Fatty acid oxidation is also impaired in PKD, and metformin can positively influence this [13]. Based on observational studies, metformin is a promising drug for early-stage ADPKD.

#### 3.4.2. Statins

Statins, or 3-hydroxy-3-methylglutaryl-coenzyme A (HMG-CoA) reductase inhibitors, possess antiproliferative, anti-inflammatory, and antioxidant properties [46]. Statins activate AMPK and have been shown to ameliorate cystic proliferation in ADPKD animal models [47]. Gile et al. [48] demonstrated that lovastatin significantly reduced cystic kidney size, cyst volume density, and serum urea nitrogen levels in heterozygous male Han:SPRD rats. A clinical trial in 110 children and young adults with ADPKD showed a benefit of pravastatin on total kidney volume [49]. However, whether statins can be a standard treatment for ADPKD requires confirmation through large-scale randomized controlled trials.

### 3.5. Anti-Inflammatory Agents

#### 3.5.1. IL-37b

Interstitial inflammation, attributed largely to macrophage infiltration, is considered a feature that exacerbates disease progression. Studies by Zylberberg et al. [50] and Yang et al. [51] demonstrated, in transgenic mice expressing human IL-37b and in early-onset ADPKD mice injected with recombinant human IL-37b, that IL-37b promotes interferon signaling in kidney-resident macrophages, thereby inhibiting cyst formation. These findings suggest IL-37 as a feasible immunomodulatory therapy for ADPKD.

#### 3.5.2. TWEAK

TWEAK is a TNF superfamily cytokine that regulates inflammatory responses. Its receptor, Fn14, is expressed on glomerular epithelial cells. Cordido et al. [52] showed that anti-TWEAK treatment slows cyst growth and improves renal function and survival in models. Anti-TWEAK antibody restored several ADPKD-related pathways (e.g., proliferation, NF-κB), mildly reduced fibrosis and apoptosis, and indirectly decreased macrophage recruitment. These results identify the TWEAK signaling pathway as a novel disease mechanism and a potential therapeutic target in ADPKD. Blocking TWEAK signaling slows cyst growth and disease progression [52].

### 3.6. MicroRNA Blockers

MicroRNAs (miRNAs) are short, non-coding RNAs that bind target mRNAs with sequence specificity, inhibiting their translation. Recent findings indicate a common set of dysregulated miRNAs in PKD mouse models, particularly the miR-17 family and miR-21, both upregulated in renal cysts and promoting ADPKD progression in mice. MiR-17 promotes cyst proliferation, while miR-21 suppresses pro-apoptotic genes, inhibiting cyst apoptosis [53]. RGLS4326 is a short oligonucleotide inhibitor of miR-17. Studies found that subcutaneous administration of RGLS4326 was safe and effective in ADPKD animal models [54]. Conversely, in later stages of cyst formation, miR-192 and miR-194 are downregulated due to hypermethylation, suggesting a potential role in limiting cyst expansion. The therapeutic effect of miR-192 and -194 precursors has been demonstrated in vivo and in vitro. Injection of miRNA precursors slowed cyst growth in Pkd1 knockout mice [55]. miRNAs have emerged as new regulators of ADPKD pathogenesis, and anti-miRs represent a viable novel drug class for treatment [53].

### 3.7. Dietary Interventions

General recommendations for ADPKD patients include a healthy diet and lifestyle, smoking cessation, maintaining optimal weight, and regular cardiovascular check-ups [1]. Additionally, salt intake should be limited to no more than 5 g per day [56].

#### 3.7.1. Caloric Restriction and Ketogenic Diet

Recently, dietary interventions such as caloric restriction (CR), time-restricted feeding (TRF), and the ketogenic diet (KD) have emerged as potential strategies to induce metabolic reprogramming and delay ADPKD progression. These interventions hold promise for controlling ADPKD by improving metabolism and reducing oxidative stress.

In animal models, Kipp et al. [57] found that CR slows cyst growth and prevents ESRD by inhibiting mTORC1 signaling. Furthermore, CR reduced the expression of pro-inflammatory cytokines (MCP-1, IL-6, TNF-α) and restored the expression of the glycolytic enzyme HK2 to normal levels. Studies show that a 30–50% reduction in caloric intake without malnutrition slows cyst growth in animal models [57,58]. Cyst cells are glucose-dependent; a sustained shift from mitochondrial oxidative phosphorylation to aerobic glycolysis is a factor driving cyst growth, making these cells sensitive to glucose availability. Preclinical studies targeting glucose metabolism via intermittent fasting are promising [42]. Torres et al. [59] experimentally confirmed that KD dramatically improved the phenotype in Han: SPRD rat models, reducing kidney weight, cyst index, and epithelial proliferation. KD also attenuated fibrosis and mTOR signaling and improved creatinine clearance. KD can also reduce kidney and cyst size in established disease, suggesting that low-calorie or ketogenic diets may slow ADPKD progression [60]. In human data, obesity is a strong independent predictor of ADPKD progression [61]. CR benefits overweight or obese patients by promoting weight loss and improving metabolic parameters [62]. KD reduces body weight and fat mass while improving glomerular filtration rate in ADPKD patients [63].

#### 3.7.2. 2-Deoxyglucose (2DG)

PKD1 mutation leads to enhanced glycolysis in the kidneys of ADPKD mice and patients [43]. Increased aerobic glycolysis becomes dominant in cyst cells. 2DG, transported into cyst cells, acts as a competitive inhibitor of the glycolytic pathway. Magistroni et al. [64] experimentally confirmed that 2DG reverses the glycolytic shift in ADPKD kidneys, improving kidney weight, volume, cyst index, and proliferation rate in ADPKD mice [65]. These studies demonstrate that metabolic regulation is a potentially important factor in ADPKD progression and a possible therapeutic target [43,66]. Multiple studies indicate 2DG is a safe and effective drug in animal models, with no toxic effects observed even with long-term, low-dose administration [67,68].

## 4. Novel Therapeutic Targets

### 4.1. Modulating Intracellular Calcium and the Cell Cycle

#### 4.1.1. TMEM16A Inhibitors

Research has found that the calcium-activated chloride channel TMEM16A is crucial for fluid secretion into renal cysts in vitro [69]. TMEM16A inhibitors reduce chloride channel activity. Niclosamide and benzbromarone, which inhibit TMEM16A, are associated with reduced cyst growth [70]. The TMEM16A-specific small molecule Ani9 also inhibits cystogenesis in vivo [71]. TMEM16A inhibitors not only block chloride current but also suppress TMEM16A expression during long-term treatment. TMEM16A inhibitors appear promising for future ADPKD therapy.

#### 4.1.2. Calcimimetics

Calcimimetics activate the calcium-sensing receptor, leading to decreased cAMP and increased intracellular calcium. The calcimimetic R568 significantly reduced kidney cyst volume in mice [72]. In ADPKD rats and mice, combined treatment with lixivaptan and the calcimimetic R568 was more effective than either alone in reducing kidney weight, cyst and fibrosis volume [73].

#### 4.1.3. Triptolide

Triptolide, a natural compound extracted from the traditional Chinese herb Tripterygium wilfordii, has been shown to restore cell membrane Ca^2+^ release and induce growth arrest in Pkd1 mouse renal epithelial cells. Leuenroth et al. [74] confirmed that triptolide reduced cyst formation from youth to adulthood in a Pkd1 ADPKD model.

#### 4.1.4. R-Roscovitine

Pharmacological inhibition of cell cycle progression using the cyclin-dependent kinase (CDK) inhibitor R-roscovitine in animal models showed that effectively inhibiting the cell cycle, proliferation, and apoptosis suppresses the progression of renal cystic disease and hepatic cystogenesis [75].

### 4.2. Histone Deacetylase (HDAC) Inhibitors

#### Benzothiazole Derivatives

Emerging evidence indicates that HDACs are important regulators in ADPKD. Cao et al. [76] designed and synthesized a series of benzothiazole-containing compounds as potential HDAC inhibitors. Given the multifaceted involvement of HDACs in cyst progression, they performed target-based screening using HeLa cell nuclear extracts to identify potent pan-HDAC inhibitors. Compound 26 emerged as the most effective candidate. Subsequent pharmacological characterization revealed it effectively inhibited multiple HDACs (especially HDAC1, HDAC2, and HDAC6, IC50 all < 150 nM), with particular sensitivity to HDAC6 (IC50 = 11 nM). This compound significantly inhibited cyst formation and expansion in an in vitro cyst model and effectively slowed cyst growth in an embryonic kidney cyst model and an ADPKD mouse model in vivo.

### 4.3. Novel Targets

#### 4.3.1. Notch3

Recent research indicates that the Notch signaling pathway plays an indispensable role in the pathogenesis of various kidney diseases, including ADPKD. Notch3 receptor is overexpressed in kidney tissues from ADPKD mice and patients. The Notch3 intracellular domain (N3ICD) and Hes1 can bind to the PTEN promoter, leading to transcriptional repression of PTEN. This further activates the downstream PI3K-AKT-mTOR pathway, promoting renal epithelial cell proliferation. γ-Secretase inhibitors, which block the proteolytic cleavage required for Notch3 activation, and shRNA-mediated knockdown of Notch3 significantly delayed renal cyst growth in vitro and in vivo. Therefore, Notch3 is a novel driver of renal epithelial cell proliferation and cyst formation in ADPKD, presenting a promising therapeutic target [77].

#### 4.3.2. AURKA

Aurora kinase A (AURKA) promotes cell proliferation and is overexpressed in different types of PKD. Tham et al. [78] deleted the Aurka gene in mouse models of ADPKD and Joubert syndrome (caused by mutations in Pkd1 and Inpp5e, respectively) and found that Aurka deletion prevented cyst formation. They also identified an association with AKT signaling in cyst prevention. Further experiments established AURKA as a master regulator of cyst development across different PKD types, functioning in part through AKT. Therefore, anti-AURKA therapy may become a new research focus.

#### 4.3.3. LncRNA

Long non-coding RNAs (lncRNAs) are a class of non-protein-coding RNAs with key functions in development and disease. Weisser et al. [79] confirmed in a new study that lncRNAs are involved in ADPKD pathogenesis. The lncRNA Hoxb3os is downregulated in ADPKD and regulates the mammalian target of rapamycin (mTOR)/Akt pathway in mouse kidneys in vivo. Reducing Hoxb3os expression in ADPKD mice activated mTOR complex 2 (mTORC2) signaling and exacerbated cyst formation.

#### 4.3.4. Glis2

Following PC1 inactivation, cyst growth requires intact primary cilia, a process termed cilia-dependent cyst activation (CDCA). Zhang et al. [80] studied kidneys from mice with cilia inactivated prior to cyst formation and identified Glis2 as a functional effector of polycystin signaling and CDCA. Changes in Glis2 expression in vitro mirrored observed CDCA changes in renal tissue, validating its association with cyst formation. In an ADPKD mouse model, Glis2 inactivation suppressed polycystic kidney disease, and pharmacological targeting of Glis2 with antisense oligonucleotides slowed disease progression, identifying Glis2 as a potential therapeutic target.

#### 4.3.5. Thiamet G

Su et al. [81] found that O-GlcNAcylation and O-GlcNAc transferase (OGT) were downregulated in kidney tissues of Pkd1-silenced mice. Furthermore, O-GlcNAcylation was shown to regulate the stability and function of the PC1 protein’s C-terminal cytoplasmic tail (CTT). Treatment of PKD mice with Thiamet G significantly reduced cystogenesis. These results reveal a unique role for O-GlcNAcylation in PKD cyst formation, positioning Thiamet G as a potential therapeutic target.

#### 4.3.6. RAGE

The RAGE gene, the receptor for advanced glycation end products, is involved in inflammatory responses and cell proliferation. Park et al. [82] confirmed that blocking RAGE function inhibits ADPKD cyst growth in vitro. Building on this, their team investigated the impact of RAGE on cyst expansion in vivo using a severe ADPKD mouse model (PC2R). Intravenous injection of adenovirus carrying anti-RAGE short interfering RNA led to RAGE knockdown, resulting in reduced kidney weight and volume. Furthermore, cystic area derived from different nephron segments decreased due to RAGE downregulation. Blood urea nitrogen and creatinine levels showed a declining trend after RAGE inhibition. Based on these results, the RAGE gene is a valid therapeutic target for ADPKD.

#### 4.3.7. Adeno-Associated Virus Serotype 1 (AAV1)

Ciobanu et al. [83] experimentally found sialic acid residues on ADPKD cyst epithelial cells that enhance transduction by adeno-associated virus serotype 1 (AAV1). AAV1 also exhibited tropism for cyst epithelium. They further observed that intervention targeting the cystic fibrosis transmembrane conductance regulator (CFTR) via AAV1 significantly reduced cyst area and volume in mice, suggesting promising prospects for AAV1 and CFTR-targeted therapy in ADPKD.

#### 4.3.8. CD8^+^ T Cells

Kleczko et al. [84] used flow cytometry to find that the numbers of CD8^+^ and CD4^+^ T cells in the kidney progressively increased with ADPKD severity, but only CD8^+^ T cells were selectively activated. Immunofluorescence analysis showed T cells specifically localized to cyst lesions, and qPCR/ in situ hybridization detected elevated levels of T cell-recruiting chemokines (CXCL9/CXCL10) in mouse kidneys, patient tissues, and ADPKD epithelial cell lines. The team also found that sustained CD8^+^ T cell immune depletion for 1–3 months in C57Bl/6 Pkd1 mice worsened ADPKD pathology, reduced apoptosis, and increased proliferation (compared to IgG controls), confirming a renoprotective role for CD8^+^ T cells. This reveals a functional role for T cells, particularly CD8^+^ T cells, in ADPKD progression. Therefore, targeting this pathway with immuno-oncology drugs may offer a novel therapeutic strategy.

#### 4.3.9. Dihydrotanshinone I (DHTS)

Mahendran et al. [85] found that dihydrotanshinone I (DHTS) inhibits ADPKD cell proliferation by inducing G1 phase cell cycle arrest and enhancing necroptosis. DHTS treatment may also modulate enhanced immune surveillance observed in untreated ADPKD cells. After elucidating related signaling pathways and ensuring safety for normal cells, the antiproliferative effect of DHTS on ADPKD cells demonstrates its potential as an alternative therapy.

#### 4.3.10. Aquaporins

Studies suggest that aquaporins—transmembrane channel proteins including AQP-1, AQP-2, AQP-3, and AQP-11—are involved in the pathogenesis of ADPKD. Aquaporins may represent potential new therapeutic targets [86].

#### 4.3.11. Super-Enhancers and Metabolic Reprogramming

Super-enhancers (SEs) are large clusters of transcriptional enhancers that drive robust expression of cell identity and disease genes. Mi et al. [87] experimentally revealed extensive SE remodeling during cystogenesis, with SE-associated transcripts most enriched in metabolic processes in cyst cells. Inhibiting the transcriptional kinase CDK7 (cyclin-dependent kinase 7), involved in SE assembly and maintenance, or targeting the CDK7-regulated metabolic target gene AMP deaminase 3 (AMPD3, slowed cyst growth in an ADPKD mouse model. The study also found that CDK7 expression is generally elevated in ADPKD patients and positively correlates with AMPD3 expression and disease severity, elucidating the mechanism by which SEs regulate metabolic gene transcription during cyst formation. Therefore, SE-driven metabolic reprogramming is a potential therapeutic target.

#### 4.3.12. Epigenetics

Zhou et al. [88] showed that DNA methyltransferase 1 (DNMT1) expression is upregulated in cystic renal epithelial cells and tissues. Knockout of the Dnmt1 gene or targeted inhibition of DNMT1 using demethylating agents delayed cyst growth in Pkd1-mutant kidneys and extended the lifespan of Pkd1 conditional knockout mice. The study also identified novel DNMT1 targets—PTPRM and PTPN22 (both belonging to the protein tyrosine phosphatase family)—which are involved in regulating the phosphorylation and activation of PKD-related signaling pathways (including ERK, mTOR, and STAT3). The researchers also found methylation dysregulation of epigenetic clock-related genes in ADPKD patients, confirming epigenetic age acceleration in their kidneys. Furthermore, the team identified five epigenetics-related genes (Hsd17b14, Itpkb, Mbnl1, Rassf5, and Plk2), with biological functions indicating their methylation status participates in ADPKD progression. Additionally, the epigenetic regulator BRD4 has been confirmed to be involved in ADPKD pathogenesis. Yang et al. [89] found that a BRD4 inhibitor exhibited high efficacy and selectivity in ADPKD cell and embryonic kidney models, potentially becoming a future therapeutic target.

#### 4.3.13. Post-Translational Modifications

Recently, post-translational modifications (PTMs) have been recognized as key factors in cystogenesis. Ren et al. [90] found that the E3 ubiquitin ligase TRAF6 is significantly upregulated in ADPKD cells, mouse models, and patient tissues. TRAF6 interacts with the transcription factor STAT3, selectively enhancing its K63-linked ubiquitination, promoting its activation and transcriptional activity, thereby cooperatively promoting renal cyst growth in ADPKD cells and mouse models. Inhibiting TRAF6 significantly suppressed STAT3 transcriptional activity and slowed cyst growth in vitro and in vivo. Therefore, targeting TRAF6 may provide a novel strategy.

Furthermore, Ren et al. [91] found that the deubiquitinase USP28, abnormally upregulated in ADPKD patients, specifically removes K48-linked polyubiquitin chains, thereby counteracting the protein degradation of signal transducer and activator of transcription 3 (STAT3). They also observed that USP28 directly interacts with and stabilizes c-Myc, a transcriptional target of STAT3. These two mechanisms synergistically promote renal cystogenesis. Pharmacological inhibition of USP28 significantly attenuated cyst formation in vitro and in vivo. Thus, USP28 participates in ADPKD pathogenesis by regulating STAT3 trafficking and its transcriptional target c-Myc, making its inhibition a potential novel strategy.

Additionally, Lv et al. [92] found that enhancer of zeste homolog 2 (EZH2) is significantly upregulated in Pkd1 cells, Pkd1 mice, and human ADPKD kidneys. Inhibiting EZH2 alleviated cyst development in MDCK cells and mouse embryonic kidney cyst models, and both conditional knockout of Ezh2 and treatment with GSK126 inhibited renal cyst growth and protected renal function in Pkd1 mice. The study showed that the cAMP/PKA/CREB pathway promotes EZH2 expression. EZH2 mediates cyst formation through three pathways: enhancing STAT3 methylation and activation, inhibiting p21 to promote the cell cycle, and activating non-phosphorylated β-catenin in the Wnt pathway. Moreover, EZH2 enhances ferroptosis in ADPKD by inhibiting SLC7A11 and GPX4. This study clarifies the critical role of EZH2 in promoting renal cyst growth via epigenetic mechanisms and suggests that inhibiting or degrading EZH2 could be a novel therapeutic strategy. All targets are summarized in the table below (Table 1).

## 5. Discussion

ADPKD, as a common inherited renal disorder, involves multiple layers of pathogenesis including loss of polycystin function, dysregulated signaling pathways, and metabolic abnormalities [2]. With deepening research into its molecular mechanisms, treatment strategies have gradually shifted from traditional symptomatic management towards targeted interventions addressing core disease processes. Based on current research, this article systematically reviews advances in ADPKD treatment and explores the potential of emerging targets, aiming to provide a theoretical foundation for clinical practice and future research.

ADPKD is primarily caused by mutations in *PKD1* or *PKD2*, leading to defective function of the polycystin complex and subsequently affecting primary cilium-mediated cell signaling. Polycystin deficiency triggers pathological changes such as disrupted intracellular calcium homeostasis, elevated cAMP levels, abnormal mTOR pathway activation, and metabolic reprogramming [7,8]. These changes collectively promote proliferation of renal tubular epithelial cells and cyst fluid secretion, ultimately forming cysts that destroy kidney structure. Furthermore, inflammatory responses and fibrosis play significant roles in disease progression. Macrophage infiltration, cytokine release (e.g., TGF-β, TWEAK), and extracellular matrix remodeling further accelerate loss of renal function. These mechanisms also provide multiple potential targets for drug development. For example, aberrant activation of the cAMP signaling pathway is a core driver of cyst growth, making cAMP pathway inhibitors like tolvaptan and somatostatin analogues hotspots for clinical research. Concurrently, metabolic abnormalities (e.g., enhanced aerobic glycolysis) and dysregulation of energy-sensing pathways (e.g., AMPK/mTOR) provide a theoretical basis for metabolic interventions like metformin and ketogenic diets.

Current pharmacological treatments for ADPKD mainly focus on: (1) cAMP pathway inhibitors; (2) mTOR signaling inhibitors; (3) AMPK agonists and metabolic modulators; (4) anti-inflammatory and immunomodulatory agents; and (5) dietary and metabolic interventions. (1) Among cAMP pathway inhibitors, tolvaptan, as the first approved targeted drug for ADPKD, delays renal function decline and kidney volume growth by antagonizing the vasopressin V2 receptor to lower cAMP levels. However, its risk of hepatotoxicity, side effects like polyuria, and high cost limit its widespread use [93,94]. Emerging therapeutic strategies beyond tolvaptan are under active investigation, including combination therapies, metabolic interventions, and gene editing. For instance, glucagon-like peptide-1 (GLP-1) receptor agonists have shown potential protective effects through metabolic modulation and anti-inflammatory actions [95,96]. Ketogenic dietary interventions have demonstrated reduced cyst growth in animal models, but clinical evidence remains limited and long-term safety concerns exist [97]. CRISPR-Cas9 gene editing has successfully corrected pathogenic mutations in in vitro models, though delivery efficiency and in vivo application require further refinement [98]. International and regional guidelines, including those from KDIGO and Taiwanese consensus statements, emphasize risk stratification using imaging, genotyping, and height-adjusted total kidney volume (htTKV), alongside individualized management [99,100,101]. Future directions integrating organoid models, systematic reviews, real-world studies, and precision medicine approaches will likely advance ADPKD care from slowing progression toward targeted repair [102,103,104]. Lixivaptan, another V2 receptor antagonist, shows similar efficacy and potentially lower hepatotoxicity in preclinical studies, but its clinical value requires further validation. Somatostatin analogues (e.g., octreotide) inhibit cAMP generation and have shown effects in slowing kidney growth in some clinical trials, but their long-term efficacy and safety need more evidence. (2) mTOR signaling inhibitors: Rapamycin and its derivatives (e.g., sirolimus) effectively inhibit cyst growth in animal models, but human clinical trial results are inconsistent, and high doses may cause adverse effects like proteinuria and hyperlipidemia. This suggests caution in using mTOR inhibitors for ADPKD; they may be more suitable for specific subgroups or combination therapies. (3) AMPK agonists and metabolic modulators: Drugs like metformin activate AMPK to inhibit CFTR and the mTOR pathway, showing potential in early-stage ADPKD patients. Statins, beyond lipid-lowering effects, can inhibit cell proliferation and inflammation via AMPK-dependent pathways. Due to their safety and accessibility, these drugs are important candidates for adjuvant therapy in ADPKD. (4) Anti-inflammatory and immunomodulatory agents: Agents like IL-37b and anti-TWEAK antibodies can reduce cyst growth and fibrosis by modulating macrophage polarization and NF-κB signaling. Additionally, MicroRNA blockers (e.g., RGLS4326) effectively inhibit cyst proliferation and promote apoptosis in animal models by targeting the miR-17 family and miR-21, opening a new direction for gene therapy in ADPKD. (5) Dietary interventions: Low-calorie diets, intermittent fasting, and ketogenic diets significantly delay disease progression in animal models by improving mitochondrial function, inhibiting glycolysis, and reducing oxidative stress. 2-Deoxyglucose (2DG), as a glycolytic inhibitor, can reverse metabolic abnormalities in cyst cells, but its long-term safety requires further evaluation.

Simultaneously, with deeper understanding of ADPKD pathogenesis, numerous novel targets have emerged. Ion channel and calcium modulators: TMEM16A inhibitors (e.g., niclosamide, Ani9) inhibit cyst expansion by blocking chloride secretion. Calcimimetics (e.g., R568) and triptolide exhibit anti-cyst effects in animal models by restoring intracellular calcium homeostasis. These drugs target the core abnormality of ion transport in ADPKD and have high translational potential. Epigenetic regulators: Histone deacetylase inhibitors (e.g., benzothiazole derivatives) can influence multiple pathogenic pathways by modulating chromatin structure. Long non-coding RNAs (e.g., Hoxb3os) and Glis2, as new epigenetic regulators, show promising effects in animal models when targeted. Furthermore, adeno-associated virus serotype 1 (AAV1)-mediated gene delivery systems offer the possibility of restoring polycystin function. Novel signaling pathway targets: Overexpression of Notch3 and AURKA promotes cell proliferation and cyst formation in ADPKD; their inhibitors or gene silencing strategies significantly delay disease progression in preclinical studies. Additionally, CDK inhibitors (e.g., R-roscovitine) and natural compounds like dihydrotanshinone I (DHTS) exhibit antiproliferative activity by inducing cell cycle arrest and apoptosis. Emerging immune and inflammatory targets: Recent studies found CD8^+^ T cells specifically accumulate in ADPKD kidneys and inhibit cyst growth through immune surveillance. This finding suggests immunomodulatory drugs (e.g., checkpoint inhibitors) could become a new therapeutic strategy. Meanwhile, RAGE receptor antagonists alleviate cyst burden in animal models by blocking inflammatory and proliferative signals.

Despite significant progress in ADPKD treatment research, many challenges remain. First, individual genetic differences demand more precise treatment strategies. Variations in mutation type (PKD1 vs. PKD2), mutation site, genetic background, and environmental factors collectively determine disease progression rate and treatment response. For example, PKD1 mutation patients typically progress faster than PKD2 patients [105], and even patients with the same mutation can have significantly different clinical presentations. Tolvaptan is effective only in rapidly progressing patients, while metabolic interventions may be more suitable for early-stage or overweight patients. Second, most novel target drugs are still in the preclinical stage, and their safety, administration routes, and long-term efficacy require validation through large-scale clinical trials. Additionally, combination therapies (e.g., tolvaptan combined with metformin may simultaneously target the cAMP pathway and metabolic abnormalities; mTOR inhibitors combined with AMPK agonists may more effectively balance cell proliferation and energy metabolism regulation) may improve efficacy and reduce side effects through multi-target synergy. Treatment for ADPKD has entered an era coexisting with targeted interventions and metabolic regulation. The continuous emergence of new targets brings new hope for ADPKD treatment. From ion channel regulation to epigenetic control, from cell cycle inhibition to immune microenvironment modulation, targets like Notch3, AURKA, and TMEM16A provide abundant options. In the future, by deeply analyzing the molecular network of ADPKD and integrating the concept of precision medicine, effective control and potentially functional cure of the disease may be achievable.

## Figures and Tables

**Table 1:** ADPKD Existing Targets And Research Results. According to the corresponding chart compiled in the previous text, it includes target category, Specific target/pathway, intervention/drug name, core Mechanism and research results.

Target Category	Specific Target/Pathway	Intervention/Drug Name	Core Mechanism	Research Status
Clinical research	RAAS Pathway (ACE/AT1 Receptor)	Angiotensin-Converting Enzyme Inhibitors (ACEI)	Block RAAS activation, reduce renal vascular resistance, slow renal disease progression; inhibit aldosterone elevation and renal parenchymal fibrosis	Routinely used clinically (for hypertension/proteinuria); monitoring required for side effects such as renal ischemia and hyperkalemia
	Vasopressin V2 Receptor	Tolvaptan	Selectively bind to V2 receptors, decrease cAMP levels in collecting ducts/thick ascending limbs of Henle’s loop, block water reabsorption, and reduce annual renal volume growth	Approved by FDA for adult ADPKD (at risk of rapid progression); some patients intolerant due to polyuria/edema
	Vasopressin V2 Receptor	Lixivaptan	Potently inhibit V2 receptors, reduce cAMP levels in cyst cells; lower hepatotoxicity risk than tolvaptan	Preclinical (animal study: >50% cyst volume reduction in ADPKD rats)
	cAMP Regulatory Pathway	Somatostatin Analogues (Octreotide, Lanreotide, etc.)	Inhibit cAMP production in liver/kidney cells, suppress fluid secretion and cell proliferation, induce apoptosis	Preclinical and preliminary clinical exploration; targeting cAMP-related pathological pathways
	mTOR Pathway	Rapamycin, Sirolimus	Inhibit mTOR enzyme activity, reduce protein translation, and suppress cell proliferation and cyst formation	Effective in animal studies (slowing rat PKD progression); insufficient safe dosage for humans; side effects include chronic proteinuria
	AMPK Pathway	Statins (Lovastatin, Pravastatin, etc.)	Activate AMPK, exert antiproliferative, anti-inflammatory, and antioxidant effects; improve cystic hyperplasia	Effective in animal studies (reducing rat cyst volume); clinical trial trends show renal protection in children/adolescents
	Inflammatory Pathway	IL-37b (Interleukin-37b)	Promote interferon signaling in renal resident macrophages and inhibit cyst formation	Validated in transgenic mouse studies; potential immunomodulatory therapy
	Inflammatory Pathway (TWEAK-Fn14)	Anti-TWEAK Antibodies	Block TWEAK signaling, restore proliferation/NF-κB pathway, reduce fibrosis, and decrease macrophage recruitment	Preclinical (slowing cyst growth and improving renal function in animal models)
	miRNA Regulation	RGLS4326 (miR-17 Inhibitor)	Inhibit miR-17 expression and block its cyst-promoting proliferative effect	Preclinically safe and effective (tolerated by subcutaneous injection in animal models)
	miRNA Regulation	miRNA-192/194 Precursors	Supplement downregulated miRNAs and inhibit cyst expansion	Validated in vitro and in vivo; not entered clinical trials
	Glycolytic Pathway	2-Deoxyglucose (2DG)	Competitively inhibit glycolytic pathway and reverse enhanced glycolysis in ADPKD kidneys	Preclinically safe and effective (improving renal weight and cyst index in mice); no obvious toxicity with long-term low-dose administration
Preclinical Research	Dietary Intervention	Caloric Restriction (CR), Time-Restricted Feeding (TRF)	Inhibit mTORC1 signaling, reduce pro-inflammatory cytokine expression, and restore normal glycolytic enzyme levels	Preclinically proven to slow cyst growth; clinically recommended as adjuvant intervention
	Dietary Intervention	Ketogenic Diet (KD)	Reduce body weight and fat mass, inhibit mTOR signaling, and improve creatinine clearance	Effective in animal studies (improving ADPKD phenotype in rats); clinically explored as metabolic intervention strategy
	Calcium Channel/Cell Cycle	TMEM16A (Calcium-Activated Chloride Channel)	Niclosamide, Benzbromarone, Ani9 (inhibitors): Inhibit chloride channel activity and reduce cyst fluid secretion	Validated in vitro and in vivo; potential clinical translation value
	Calcium-Sensing Pathway	Calcimimetics (R568)	Activate calcium-sensing receptors, decrease cAMP, and increase intracellular calcium concentration	Effective as monotherapy in animal studies; synergistic effect with lixivaptan
	Cell Membrane Ca^2+^ Regulation	Triptolide	Restore cell membrane Ca^2+^ release and induce growth arrest of renal epithelial cells	Preclinical (reducing cyst formation in Pkd1 mice)
	Cell Cycle (CDK)	R-roscovitine (CDK Inhibitor)	Inhibit cell cycle progression, reduce cell proliferation and apoptosis, and suppress renal/hepatic cyst formation	Preclinically effective (slowing cyst disease progression in animal models)
	Histone Deacetylases (HDACs)	Benzothiazole Derivatives (Compound 26)	Potently inhibit HDAC1/2/6 and suppress cyst formation and expansion	Validated in vitro and in animal models (IC50 < 150 nM); not entered clinical trials
	Notch3 Pathway	γ-Secretase Inhibitors, Notch3 shRNA	Block Notch3 activation, relieve its transcriptional inhibition of PTEN, and suppress PI3K-AKT-mTOR pathway	Validated in vitro and in vivo to slow cyst growth; novel driver target
	Aurora Kinase A (AURKA)	AURKA Gene Knockout/Inhibitors (Under Development)	Inhibit AURKA-mediated cell proliferation and block AKT signaling transduction	Preclinical (preventing cyst formation in PKD mouse models); potential research hotspot
	Long Non-Coding RNA (lncRNA)	Hoxb3os Modulators (Under Development)	Regulate Hoxb3os expression and inhibit mTORC2 signaling transduction	Preclinically proven to be associated with cyst formation; targeted intervention alleviates disease
	Cilia-Dependent Cyst Activation (CDCA)	Glis2 Antisense Oligonucleotides	Inhibit Glis2 expression, block CDCA process, and suppress cyst growth	Preclinical (slowing ADPKD progression in animal models)
	O-Glycosylation (OGT)	Thiamet G	Upregulate O-GlcNAcylation, stabilize PC1 protein function, and reduce renal cell production	Validated in PKD mouse models; novel metabolic modification target
	Receptor for Advanced Glycation End Products (RAGE)	Anti-RAGE siRNA (Adenoviral Vector)	Downregulate RAGE gene, inhibit inflammation and cell proliferation, and reduce cyst area	Preclinically effective (decreasing renal weight/volume and improving renal function indices)
	Cyst Epithelium-Targeted Delivery	Adeno-Associated Virus Type 1 (AAV1) + CFTR Intervention	AAV1 tropism for cyst epithelium; combined with CFTR regulation to reduce cyst area and volume	Preclinically validated; targeted delivery system under exploration
	Immune Regulation	CD8^+^ T Cell Activators (Under Development)	Enhance renoprotective effects of CD8^+^ T cells, inhibit cyst proliferation, and promote apoptosis	Preclinically proven CD8^+^ T cell function; immuno-oncology strategy under exploration
	Cell Cycle/Immune Surveillance	Dihydrotanshinone I (DHTS)	proliferation, and promote apoptosis Induce G1 phase cell cycle arrest, enhance necrosis, and regulate immune surveillance	In vitro proven antiproliferative effect on ADPKD cells; safety validation in normal cells required
	Aquaporins (AQP1/2/3/11)	Aquaporin Inhibitors (Under Development)	Block aquaporin-mediated water transport and inhibit cyst fluid accumulation	Mechanistic studies confirm involvement in pathogenesis; potential novel target with no specific drugs available
	Super-Enhancers (SEs)/Metabolic Reprogramming	CDK7 Inhibitors, AMPD3 Inhibitors	Inhibit SE assembly or their regulated metabolic target genes, and slow cyst growth	Preclinically effective; CDK7/AMPD3 expression positively correlated with disease severity in ADPKD patients
	Epigenetic Regulation	DNMT1 Inhibitors (Demethylating Agents)	Downregulate DNMT1, restore PTPRM/PTPN22 expression, and inhibit ERK/mTOR/STAT3 pathways	Prolonged survival and slowed cyst growth in Pkd1-knockout mice
	Epigenetic Regulation (BRD4)	BRD4 Inhibitors (Under Development)	Inhibit BRD4-mediated transcriptional regulation and block cyst formation	Highly selective and effective in cell and embryonic kidney models; potential target
	Post-Translational Modification (Ubiquitination)	TRAF6 Inhibitors (Under Development)	Inhibit TRAF6-mediated K63 ubiquitination and activation of STAT3, and reduce cyst growth	Validated in vitro and in vivo
	Post-Translational Modification (Deubiquitination)	USP28 Inhibitors (Under Development)	Inhibit USP28-mediated stabilization of STAT3/c-Myc and block cyst formation	Significantly reduced cystogenesis in vitro and in vivo
	Post-Translational Modification (Methylation)	EZH2 Inhibitors (GSK126, etc.)	Inhibit EZH2-mediated STAT3 methylation, Wnt pathway activation, and enhance ferroptosis	Inhibited cyst growth and preserved renal function in Pkd1delta/delta mice

## Data Availability

No new data were created or analyzed in this study. Data sharing is not applicable to this article.

## Abbreviations

The following abbreviations are used in this manuscript:

ADPKD	Autosomal dominant polycystic kidney disease
PC1	Polycystin-1
PC2	Polycystin-2
ESRD	End-stage renal disease
VEGF	Vascular endothelial growth factor
PI3K	Phosphoinositide 3-kinase
NFAT	Nuclear factor of activated T-cells
ECM	Extracellular matrix
ERK	Extracellular signal-regulated kinase
RAAS	Renin–angiotensin–aldosterone system
ACEIs	Angiotensin-Converting Enzyme Inhibitors
cAMP	Cyclic adenosine 3′,5′-monophosphate
FDA	Food and Drug Administration
CFTR	Cystic fibrosis transmembrane conductance regulator
PIKK	PI3K-related kinase
CR	Caloric restriction
TRF	Time-restricted feeding
KD	Ketogenic diet
CDK	Cyclin-dependent kinase
AURKA	Aurora kinase A
lncRNAs	Long non-coding RNAs
AAV1	Adeno-Associated Virus Serotype 1
DHTS	Dihydrotanshinone I
DNMT1	DNA methyltransferase 1
PTMs	Post-translational modifications
